# Limits in the use of cPTIO as nitric oxide scavenger and EPR probe in plant cells and seedlings

**DOI:** 10.3389/fpls.2013.00340

**Published:** 2013-08-29

**Authors:** Stefano D’Alessandro, Bianca Posocco, Alex Costa, Georgia Zahariou, Fiorella Lo Schiavo, Donatella Carbonera, Michela Zottini

**Affiliations:** ^1^Department of Biology, University of PadovaPadova, Italy; ^2^Department of Chemical Sciences, University of PadovaPadova, Italy; ^3^Department of Biosciences, University of MilanMilano, Italy

**Keywords:** plant, nitric oxide, cPTIO, electron paramagnetic resonance (EPR), NO scavenger, DAF-FM, *Arabidopsis*

## Abstract

Over the last decade the importance of nitric oxide (NO) in plant signaling has emerged. Despite its recognized biological role, the sensitivity and effectiveness of the methods used for measuring NO concentration in plants are still under discussion. Among these, electron paramagnetic resonance (EPR) is a well-accepted technique to detect NO. In the present work we report the constraints of using 2-4-carboxyphenyl-4,4,5,5-tetramethylimidazoline-1-oxyl-3-oxide (cPTIO) in biological samples as spin trap for quantitative measurement of NO. EPR analyses on *Arabidopsis* cell cultures and seedlings show that cPTIO(NNO) is degraded in a matter of few minutes while the (INO) compound, produced by cPTIO and NO reaction, has not been detected. Limitations of using this spin trap in plant systems for quantitative measurements of NO are discussed. As NO scavenger, cPTIO is widely used in combination with 4-amino-5-methylamino-2^′^,7^′^-difluorofluorescein (DAF-FM) fluorescent dye in plant research. However, the dependence of DAF-FM fluorescence on cPTIO and NO concentrations is not clearly defined so that the range of concentrations should be tightly selected. In this context, a systematic study on cPTIO NO scavenging properties has been performed, as it was still lacking for plant system applications. The results of this systematic analysis are discussed in terms of reliability of the use of cPTIO in the quantitative determination and scavenging of NO in plants and plant cultured cells.

## INTRODUCTION

Nitric oxide (NO) is a signal molecule involved in controlling both physiological processes and stress responses (Mur et al., 2013). It plays an important role in root organogenesis and development (Correa-Aragunde et al., 2004) and in auxin signaling (Kramer and Bennett, 2006) and perception (Terrile et al., 2012). In response to pathogen attacks, NO turns to be a key molecule in the hypersensitive response (HR) and programmed cell death (PCD) events (Wang et al., 2013). Recently, the role of NO has also been investigated in abscisic acid (ABA)-associated response of guard cells to pathogens (Ye et al., 2013).

The central role of NO in plants is corroborated by the presence of many different enzymatic and non-enzymatic sources (Gupta et al., 2010). However, the controversial existence of NO synthase-like enzymes makes it difficult to define the specific NO source engaged in a specific physiological process and to understand how it is involved in it. For this reason, in order to establish whether and where NO is produced by specific cells and tissues, plant researchers rely on several indirect methods of analysis. Many of the methods developed for NO detection capitalize on its high diffusibility as well as on its broad spectrum of chemical reactivity. However, in biological systems, the use of these methods is limited by the short half-life of the molecule (Woldman et al., 1994; Gupta and Igamberdiev, 2013).

Electron paramagnetic resonance (EPR) is a well-accepted spectroscopic technique to detect NO in a liquid phase (Hogg, 2010). This technique is selective for monitoring radical species. In principle, being NO a radical, a direct measurement by EPR should be possible; however, due to its fast spin relaxation time, it cannot be detected. Therefore, the methods of NO detection in solution through EPR are based on the trapping of NO with the formation of stable paramagnetic species (Hogg, 2010). As a matter of fact, in biological samples spin trapping methods are largely used for detection of short-living radicals such as $O_{2}^{-}$, OH^•^, both *in vivo* and *in vitro* (Berliner, 2000). Spin trapping is necessary since conventional EPR requires a steady state concentration of the free radical higher than 0.01 μM.

Iron dithiocarbamates have been widely used as spin traps, due to their high affinity for NO. The formation of stable nitrosyl iron-dithiocarbamate complexes gives a three-line EPR spectrum at room temperature, characterized by the hyperfine interaction with the N nucleus of NO (Vanin et al., 2000). However, the use of iron dithiocarbamates is problematic for quantitative NO determination, either *in planta* or in cultured cells, due to the interference of nitrites and nitrates that can produce NO under the reducing experimental conditions required for this assay (Hogg, 2010).

Alternatively, nitroxide spin traps have been tested *in vitro* and in animal cell systems (Haseloff et al., 1997). A well-known nitroxide spin trap for NO used in biological samples is 2-4-carboxyphenyl-4,4,5,5-tetramethylimidazoline-1-oxyl-3-oxide (cPTIO) that belongs to the nitronyl nitroxides (NNO) compounds. NNOs are stable organic radicals that react with NO, with rate constant of about 10^4^ M^-^^1^ s^-^^1^, forming imino nitroxides (INO) with a significant change in the associated EPR spectra (Yoshioka et al., 1996). In fact, following this reaction, the number of lines in the EPR spectra changes from five to seven.

For its chemical properties cPTIO has been commonly used also as a NO scavenger in combination with 4-amino-5-methylamino-2′, 7′-difluorofluorescein (DAF-FM) fluorescent dye, although many pitfalls have been evidenced (Vitecek et al., 2008; Rumer et al., 2012). Conversion of DAF-FM to the corresponding triazole forms (DAF-FM-T) by reaction with NO causes little changes in the absorbance maxima but greatly increases the fluorescence quantum efficiency. DAF-FM dyes react with N_2_O_3_, a by-product of NO oxidation, with a resulting increase in fluorescence, dependent on NO concentration. cPTIO is used as a scavenger of NO, to remove the increase of DAF-FM fluorescence, and prove in this way the production of NO in the system. However, it has also been shown that cPTIO, under particular experimental conditions, may facilitate formation of N_2_O_3_ by increasing the rate of NO oxidation, thus inducing an increase, instead of a decrease, of DAF-FM fluorescence (Arita et al., 2006). In fact, cPTIO oxidizes NO forming ^•^NO_2_ radical (NO + cPTIO → ^•^NO_2_ + cPTI), which in turn can react with NO to form N_2_O_3_ (NO_2_ + NO → N_2_O_3_). The sensitivity of the fluorescence intensity to pH and ascorbic acid was also considered as a source of uncertainty in the detection of NO in plants.

Despite these intrinsic problems, the advantages of cPTIO to be specific for NO and cell permeable (Vitecek et al., 2008), along with its widespread use in plant experiments, prompted us to perform a systematic study on cPTIO NO scavenging properties, since a detailed analysis was still lacking regarding applications to plant systems.

## RESULTS

### cPTIO AS A SPIN TRAP FOR NO DETECTION IN PLANTS

**FIGURE 1:**
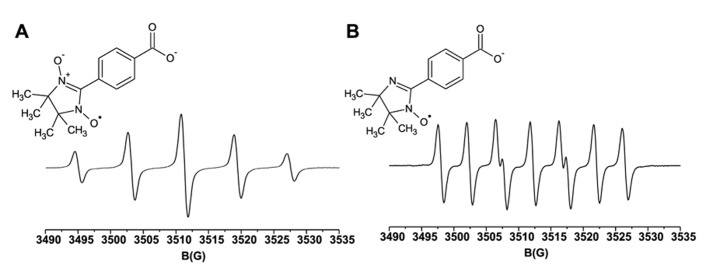
**Molecular structures and room temperature EPR spectra.(A)** 100 μM cPTIO(NNO); **(B)** 100 μM cPTIO(INO), in water.

We have evaluated the use of cPTIO as NO spin trap in plants by analyzing its EPR spectrum in different experimental conditions. In **Figure 1**, the reference spectrum of 100 μM cPTIO(NNO) in water is shown. Based on the stoichiometry of the reaction between cPTIO and NO (1:2) (Hogg et al., 1995) and on the NO release stoichiometry by the NO donor ((Z)-1-(*N*-Methyl-*N*-[6-(*N*-methylammoniohexyl)-amino])-diazen-1-ium-1,2-diolate) MAHMA NONOate, 200 μM of the NO donor was used to obtain a saturated signal corresponding to about 100 μM cPTIO(INO), whose EPR spectrum is also shown in **Figure 1**. In line with previous literature, cPTIO(NNO) gives a five-line EPR spectrum, characterized by hyperfine splitting due to the presence of two equivalent N nuclei, while cPTIO(INO) shows the specific seven-peak spectrum due to the presence of two non-equivalent N nuclei.

**FIGURE 2:**
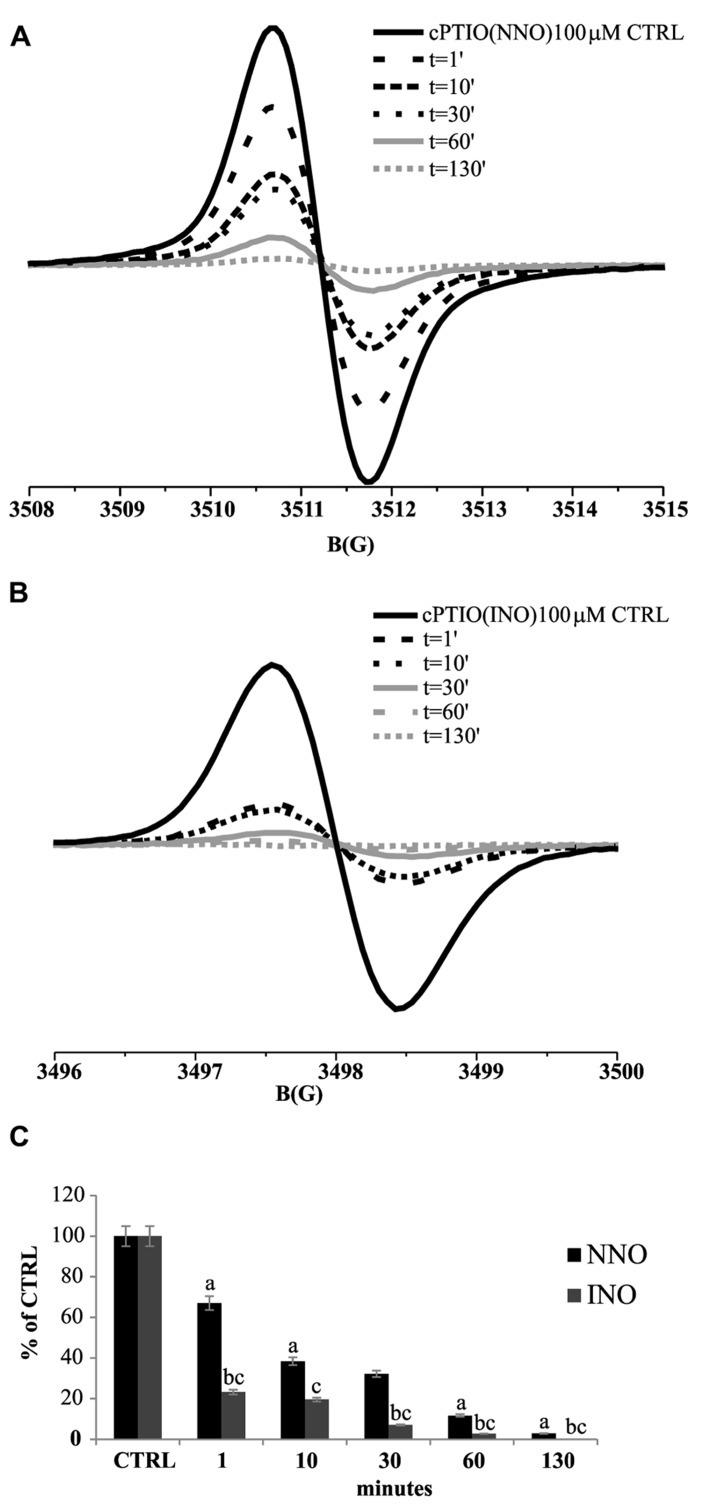
**Time dependence of cPTIO(NNO) and cPTIO(INO) EPR signals in suspension cultured cells.** Central line of cPTIO(NNO) **(A)**, and low field, first line of cPTIO(INO) **(B)** EPR spectra were quantified. In **(C)** the intensity of EPR signal for each measurement, is presented as percentage of the total signal resulting from measurement of 100 μM cPTIO(NNO) or (INO) dissolved in water (CTRL) ± SD. cPTIO(NNO) or (INO) was added to the supernatant of 5-day-old *Arabidopsis* suspension cultured cells and aliquots of the medium were collected at the time points indicated. A Student’s *t*-test was performed for each experiment and statistically significant data are marked: (a) *p* < 0.01 cPTIO(NNO) compared with the previous time point, (b) *p* < 0.01 cPTIO(INO) compared with the previous time point, (c) *p* < 0.01 cPTIO(INO) compared with cPTIO(NNO) at the same time point.

To assess the spin trap stability in the presence of biological samples, a series of experiments were performed *in vivo* on *Arabidopsis* cultured cells, by incubating 5-day-old cell cultures with 100 μM cPTIO(NNO) or cPTIO(INO). The EPR measurements were done on the culture medium after different incubation times (from 1 to 130 min). It was observed that the intensity of EPR signals of both cPTIO(NNO) and cPTIO(INO) rapidly decreased in the first minutes of incubation, reaching nearly zero after 130 min (**Figure 2**). The disappearance of cPTIO(NNO) signal was not followed by the appearance of cPTIO(INO) spectrum.

**FIGURE 3:**
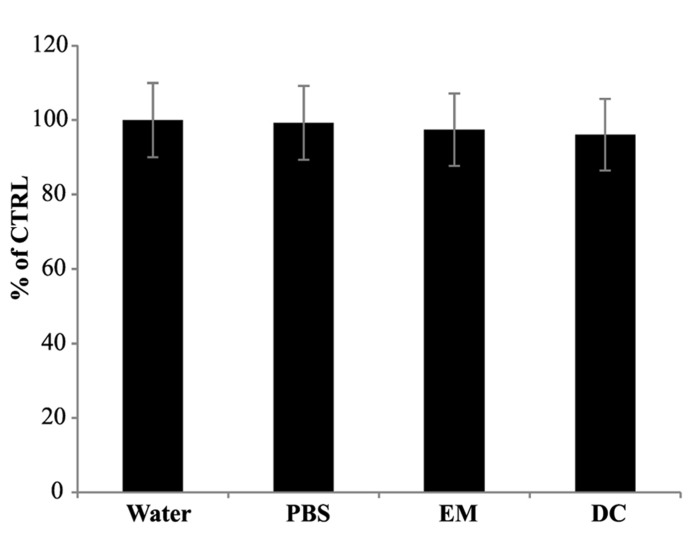
**Electron paramagnetic resonance signal of cPTIO incubated in water, PBS, exhausted medium or with dead cells.** cPTIO(NNO) was incubated in water, PBS, exhausted medium (EM) or with boiled dead cells (DC). EPR spectra of the samples were detected after 180 min of incubation. Intensities of EPR signals are given as percentage of the total signal at *t*_0_.

In order to verify whether the reduction of cPTIO EPR signal was associated with the presence of a cell-linked activity, EPR measurements were performed incubating cPTIO(NNO) either in exhausted culture medium, withdrawn from 5-day-old cell cultures, or in the presence of boiled 5-day-old cell cultures. In both cases, the intensity of EPR signals was maintained for longer time compared with the previous experiments, with a signal decrease of less than 10% after 180 min (**Figure 3**).

Two hypotheses can explain why the intensity of EPR signals rapidly decreases in cell cultures. The first is a fast uptake of cPTIO, which accumulates inside the cells, so that it becomes not measurable in the culture medium; the second is that cPTIO is rapidly transformed in an EPR silent product, either inside, after uptake, or outside the cells.

**FIGURE 4:**
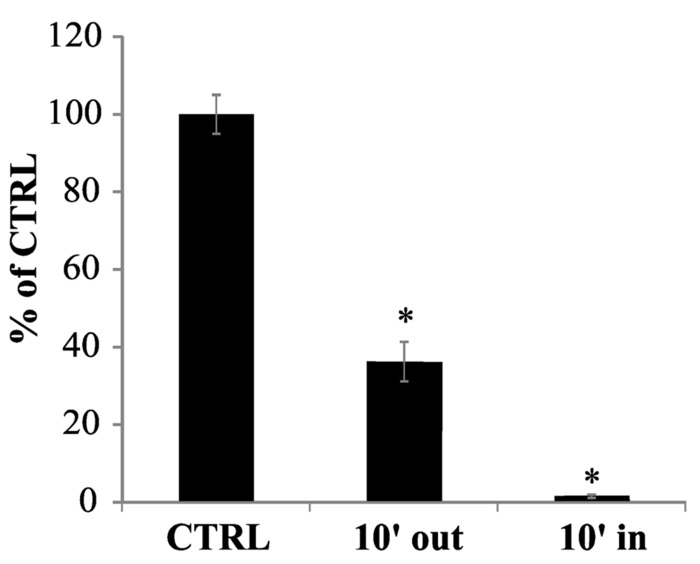
**cPTIO uptake in cell cultures.** cPTIO(NNO) was incubated with cell cultures. At 10 min a sample from the culture medium was collected (10′ out) and all cells were harvested. The cells were disrupted and centrifuged. An aliquot of the supernatant was collected (10′ in). EPR signals were detected and given as percentage of the total signal resulting from measurement of 100 μM cPTIO(NNO) dissolved in water (CTRL) ± SD. A Student’s *t*-test was performed and samples statistically different from the CTRL marked with an asterisk

To clarify this point, 100 μM cPTIO was incubated with *Arabidopsis* cell cultures for 10 min. After this time, the cells were separated from the medium and the EPR signal was measured both in the medium and in the total soluble cell extract. The intensity of the EPR signal measured in the external medium significantly decreased after 10 min of incubation (**Figure 4**). A small cPTIO(NNO) signal was detected also in the cell extract, showing that cPTIO was actually entering the cells but its concentration resulted strongly reduced when compared to the bulk concentration initially added to the sample (about 1% of the signal of 100 μM cPTIO in water; **Figure 4**). This result proves that the decrease of the EPR signal observed in the medium is not due to the spin trap accumulation inside the cells, but rather to the disappearance of cPTIO.

The degradation of cPTIO(INO) by the cells, at a faster rate compared to that of cPTIO(NNO) (**Figure 2**), implies that, for an *in vivo* quantitative measurement of NO via EPR, the use of cPTIO is not feasible because cPTIO(INO) is not stable and does not accumulate in a steady state concentration reaching the sensitivity of the EPR technique. On the other hand, the fact that 100 μM cPTIO(NNO) disappears in a short time (with a decay time constant of about 15 min^1^) and in a measurable way indicates that the endogenous NO, present in low concentration, is not the main responsible for the reactions undergone by cPTIO(NNO). This hypothesis was also supported by a series of experiments on cultured cells treated with salicylic acid (SA), which induces an increase of NO production (Zottini et al., 2007), to evaluate influence of NO on the decay rate of cPTIO(NNO) EPR signal. In that instance, it was found that the decay rate of cPTIO(NNO) was not affected by the treatment (not shown) meaning that the main reason for cPTIO(NNO) disappearance was not the reaction with NO but with other substrates such as reductans present in the cells (Haseloff et al., 1997). Thus, both the fast transformation of cPTIO(INO) and the competitive reactions of cPTIO(NNO) with substrates others than NO contribute to hinder the quantification of NO in living cells by using cPTIO as spin trap.

### cPTIO NO SCAVENGING EFFICACY IN *IN VIVO* MEASUREMENTS

**FIGURE 5:**
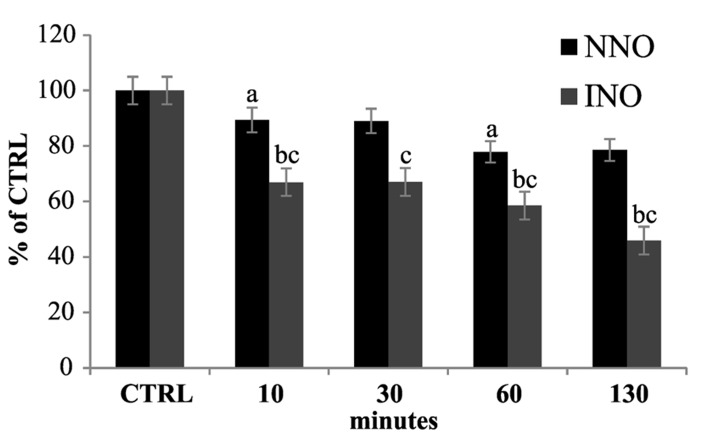
**Time dependence of cPTIO EPR signals in seedlings.** 8-day-old *Arabidopsis* seedlings were incubated in 50 ml of liquid culture medium. cPTIO(NNO) or (INO) was added to the supernatant and aliquots of the medium were taken at indicated time points. EPR measurements are presented as percentage of the total signal resulting from measurement of 100 μM cPTIO(NNO) and (INO) dissolved in water (CTRL) ± SD. A Student’s *t*-test was performed for each experiment and statistically significant data marked: (a) *p* < 0.01 cPTIO(NNO) compared with the previous time point, (b) *p* < 0.01 cPTIO(INO) compared with the previous time point, (c) *p* < 0.01 cPTIO(INO) compared with cPTIO(NNO) at the same time point.

cPTIO is widely used as NO scavenger in plant experimental systems to validate the involvement of NO in pathways triggered by different external/internal stimuli. cPTIO is used in plant cell cultures but also in experiments carried out on plant seedlings. Therefore, the kinetics of cPTIO reactions was also examined in this experimental system. The experiments were performed on *Arabidopsis* 8-day-old seedlings, incubated in 50 ml of liquid medium, by adding 100 μM cPTIO(NNO) to the external medium. The EPR measurements were performed on the culture medium after several incubation times (from 1 to 130 min). As shown in **Figure 5**, a decrease of EPR signal associated to cPTIO was observed, but it was slower when compared to that of cell cultures. A possible explanation for different decreasing rates could be the much more complex and slower process of cPTIO uptake in the whole plant compared to cultured cells. Thus, the uptake may become a rate-determining step in the cPTIO EPR signal disappearance. The decrease in the EPR signal of cPTIO was not accompanied by the formation of the INO EPR signal in cell cultures, as well as in seedlings.

**FIGURE 6:**
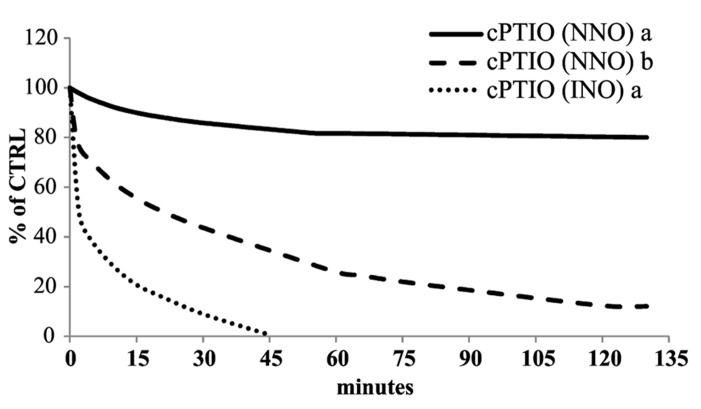
**Time dependence of cPTIO EPR signals in *Arabidopsis* total soluble extract.** 100 μM cPTIO(NNO) or (INO) was incubated in 50 mM PBS pH 7 in the presence of a protein concentration of (a) 0.3 mg/ml or (b) 1.8 mg/ml *Arabidopsis* total extract. The time course of the reactions was followed as decrease of the EPR signals. The EPR signals for each measurement were presented as percentage of the total signal resulting from measurement of 100 μM cPTIO dissolved in PBS. The plot reported is representative of three independent experiments.

The experiments performed on boiled cell culture reported above indicated that the disappearance of the cPTIO EPR signal was dependent on a cell-linked activity. To validate this hypothesis the stability of cPTIO incubated with different amount of *Arabidopsis* total soluble extract was investigated. **Figure 6** shows the time dependence of the 100 μM cPTIO(NNO) and (INO) EPR signals following the addition of different amounts of extract. The intensity of EPR signals strongly decreased depending on both the incubation time and the extract concentration. This result strongly supports an enzyme-dependent transformation of the chemical compounds. cPTIO(INO) showed a faster decay rate compared to cPTIO(NNO), using the same concentration of total extract. Moreover the EPR signal of cPTIO(INO) in the presence of the higher concentration of total soluble extract (1.8 mg/ml) was not even detectable (data not shown).

A common method for NO detection is the use of DAF-FM fluorescent dye and its membrane-permeable diacetate form (Kojima et al., 1998). DAF-FM has been used to localize NO production site in plant cells and tissues (Correa-Aragunde et al., 2004), and quantify the production of NO in suspension cultured cells (Krause and Durner, 2004). In each of these studies, fluorescence quenching by cPTIO has been used as confirmation that DAF-FM fluorescence was indeed due to NO (Gupta and Igamberdiev, 2013), since cPTIO is known to be acting as a specific NO scavenger. However, it has been shown that in the presence of high levels of NO, cPTIO can induce an increase of DAF-FM fluorescence, rather than a quenching, through a complex pathway of oxidation reactions (Vitecek et al., 2008).

The experimental data reported here have proven that cPTIO is rapidly transformed in an EPR silent compound in samples containing cells or seedlings. Thus, it is important to understand whether the reaction products of cPTIO are still able to scavenge NO. To evaluate this, cell cultures stimulated by SA were pretreated for different incubation times with cPTIO, and NO was detected by DAF-FM. In a previous paper (Zottini et al., 2007), NO production induced by SA in *Arabidopsis* cell cultures has been already reported and in that case it was measured with DAF-FM and oxyhemoglobin, in parallel. The two techniques showed indeed comparable results, confirming NO production triggered by SA.

**FIGURE 7:**
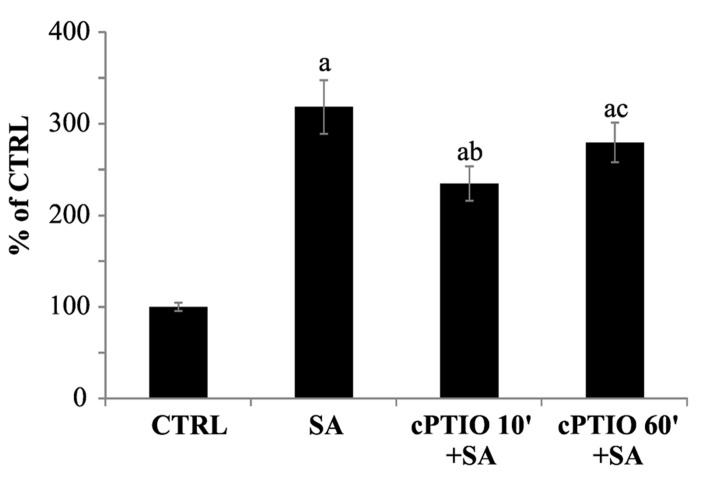
**Time dependence of DAF-FM fluorescence quenching by cPTIO.** 100 μM cPTIO(NNO) was added to cell cultures and kept for different incubation times (10 or 60 min) before 1 mM salicylic acid (SA) addition. NO levels were analyzed 60 min after SA treatment by using DAF-FM (excitation: 488 nm, detection: 515 nm). Signal from 3D reconstruction was quantified by image densitometry and reported as percentage of the not treated sample (CTRL) ± SD. A Student’s *t*-test was performed for each experiment and statistically significant data marked: (a) *p* < 0.01 compared with CTRL, (b) *p* < 0.01 compared with SA, (c) *p* < 0.01 compared with cPTIO 10′+SA.

As reported in **Figure 7**, the increasing of cPTIO incubation time leads to a reduction of its scavenging efficacy. While cPTIO pre-incubated for 10 min is able to scavenge SA-induced NO, a longer pre-incubation significantly decreases the scavenging efficacy. These results demonstrate that molecules deriving from cPTIO cell reactions are not able to scavenge NO.

## DISCUSSION

In the present work, we provide a systematic study to evaluate the efficacy of using cPTIO as NO spin trap and NO scavenger in plant systems, in particular, in cell cultures and seedlings.

The nitronyl nitroxides have been already used *in vitro* and in animal systems as spin traps for NO (Woldman et al., 1994; Haseloff et al., 1997) because of their specificity for NO compared to other spin traps, such as iron dithiocarbamates or oxyhemoglobin (Hogg, 2010).

It has been reported that in those systems cPTIO(NNO) and (INO) are transformed in the EPR silent form hydroxylamine (Woldman et al., 1994; Haseloff et al., 1997). The occurring reaction is likely a reduction associated to the presence of reducing substrates, such as glutathione and/or ascorbate, in the cell environment. Superoxide has also been reported as a possible reductant of nitroxides (Haseloff et al., 1997).

Our results strongly suggest that, also in plant cells, different reducing species may react with cPTIO. Therefore, the use of nitronyl nitroxides as spin traps for NO detection via EPR in plant systems, where endogenous rates of NO generation are very low, is compromised by their very rapid reduction into diamagnetic EPR silent products.

We also evidenced that the reduction of cPTIO is an enzyme-mediated process. In fact, it was observed that cPTIO(NNO) and cPTIO(INO) EPR signals did not decrease in fresh culture medium (data not shown), in exhausted medium, or in boiled cell suspensions. As expected, their decay rates increased after the addition of cellular extract.

Summarizing, the competitive reactions of cPTIO, and the fast reduction of cPTIO(INO), make the use of cPTIO as spin trap for NO detection via EPR unmanageable, at least in the micromolar range of NO concentrations.

The other question addressed was whether the use of cPTIO as NO scavenger was reliable, in spite of all the occurring transformation events. To shed light on this controversial point, we carried out a series of experiments with plant cells and seedlings. The results clearly indicate that also the scavenging abilities of cPTIO may be impaired due to cellular reactions. Actually, we observed that when NO production was induced by SA, the scavenging efficacy of 100 μM cPTIO was significantly reduced in a time-dependent manner. We, thus, infer that to obtain a strong scavenging effect, a higher concentration of cPTIO should be used. On the other hand, it has to be kept into consideration that high concentrations of cPTIO can give rise to artifacts, when DAF-FM is used as detection method (Arita et al., 2006).

In conclusion, the reported analysis underlines the drawbacks of using cPTIO as EPR probe for *in vivo* measurements of NO in plants. In addition, the results provide helpful indication for the right use of cPTIO as NO scavenger. In fact, in order to effectively scavenge NO, the parallel depletion of cPTIO in living cells has to be taken into account. The relatively low cPTIO concentration used in our experiments has allowed us to evidence better the time dependence of cPTIO degradation, confirming data obtained by EPR measurements. At the same time, it is evidenced that the use of low concentration of cPTIO could compromise its scavenging efficacy due to competitive reactions.

The complex chemical behavior of cPTIO in plant environment may explain why cPTIO is not always able to completely scavenge NO, especially for treatments inducing a gradual and continuous production of NO.

Since cPTIO is highly specific to NO, its use remains valuable. However, to produce significant data, and observe the scavenging effect of cPTIO, concentrations and incubation time should be accurately chosen, depending on the analyzed system and in relation to the amount of NO produced.

## MATERIAL AND METHODS

### CHEMICALS

2-4-Carboxyphenyl-4,4,5,5-tetramethylimidazoline-1-oxyl-3-oxide (Alexis Biochemicals ALX-430-001), DAF-FM-DA (Alexis Biochemicals, ALX-620-071), SA (S7401 SIGMA), MS medium salt including vitamins (Duchefa M 0409), MAHMA NONOate (Alexis, Vinci, Italy).

### CELL CULTURES

Suspension cell culture was generated from hypocotyls dissected from young plantlets of *Arabidopsis* (ecotype *Landsberg erecta*) and subcultured in AT3 medium (Desikan et al., 1996). For subculture cycles, 5 ml of cell culture volume [0.8 g fresh weight (FW)] was placed in 100 ml Erlenmeyer flasks containing 45 ml of liquid medium. Cells were subcultured in fresh medium at 7 days intervals and maintained in a climate chamber on a horizontal rotary shaker (80 rpm) at 24°C with a 16-/8-h photoperiod and a light intensity of 70 mmol m^-^^2^ s^-^^1^. All analyses and treatments with filter-sterilized solutions of SA were carried out with 5-day-old cultures (4g FW).

### *Arabidopsis* SEEDLINGS

Seeds of *Arabidopsis* (ecotype *Columbia*) were surface sterilized by washing with 70% EtOH, 0.05% Triton X 100. After the sterilization they were grown on MS – ½ medium supplemented with 0.5 g/l MES-KOH pH 5.7, 0.8% plant agar, and 1% sucrose. After 48 h of incubation at 4°C in the dark, plates were put in a growing chamber at 22°C and long day light period (16 h light/ 8 h dark). The plates were kept vertically. Seedlings of 8 days were used for the experiments (4 g FW).

### *Arabidopsis* TOTAL SOLUBLE EXTRACT

100 mg of *Arabidopsis* cells or seedlings were homogenized by Eppendorf micropestle in extraction buffer added 1:1 w/v (50 mM PBS pH 7, EDTA 1 mM, protease inhibitor cocktail). The samples were centrifuged 1 min at 16000 × *g* at 4°C. The supernatant was recovered and quantified by Bradford protein assay test (Biorad). A protein content of 0.3 or 1.8 mg/ml was used in each experiment.

### cPTIO ANALYSES

100 μM cPTIO(NNO) or cPTIO(INO) was added directly to the *Arabidopsis* cells culture. Aliquots of the medium were collected at different incubation time, and immediately frozen in liquid nitrogen. Samples were then analyzed by EPR spectroscopy at room temperature, after thawing.

The experiments with boiled dead cells were performed using *Arabidopsis* cell cultures, boiled for 30 min.

The experiments with exhausted medium were performed incubating 100 μM cPTIO(NNO) in the medium withdrawn from 5-day-old cell cultures.

8 days-old *Arabidopsis* seedlings (4 g FW) were incubated in 50 ml liquid culture medium (MS – ½ medium supplemented with 0.5 g/l MES-KOH pH 5.7, 0.8% plant agar, and 1% sucrose). 100 μM cPTIO was added to the medium. Aliquots of the medium were analyzed by EPR.

100 μM cPTIO was incubated with 0.3 or 1.8 mg/ml total soluble extract concentration, diluted in PBS and added to the capillary for EPR measurements.

The EPR signals for each measurement were presented as percentage of the total signal resulting from measurement of 100 μM cPTIO dissolved in water.

### STATISTICAL ANALYSES

All experiments were performed at least three times on independent biological replicates. The results are presented as mean ± SD (standard deviation). Statistical differences were determined by using Student’s *t*-test. Statistical significance was assigned at *p* < 0.01.

### DAF-FM ANALYSES

2-4-Carboxyphenyl-4,4,5,5-tetramethylimidazoline-1-oxyl-3-oxide was added to different flasks of *Arabidopsis *cell culture of 5 days (4 g FW) and incubated for different pre-incubation times. After the cPTIO pre-incubation 15 μM DAF-FM-DA was loaded in the cells as previously described (Zottini et al., 2007). 1 mM SA was added to the cell culture and cells were analyzed after 60 min of treatment. Samples were observed by confocal microscopy using the 488 Argon line for excitation. 3D reconstruction of the cells were obtained by Nikon PCM2000 (Biorad) laser scanning confocal microscope. DAF-FM Fluorescence was quantified by image densitometry analysis of the pixel intensities using ImageJ software (NIH, USA). At least 20 cells per samples were singularly analyzed.

### EPR SPECTROSCOPY

Room temperature continuous wave EPR spectra were collected using a Bruker Elexsys E580-X-band spectrometer equipped with the Elexsys Super High Sensitivity Probehead. All measurements were performed in capillaries (ID 0.9 mm; 50 μl total volume). Acquisition parameters were the following: microwave frequency = 9.86 GHz; modulation amplitude in the range 0.15–0.3 Gauss, microwave power = 6.370 mW; sweep time 167.77 s, time constant 40.96 ms.

## Conflict of Interest Statement

The authors declare that the research was conducted in the absence of any commercial or financial relationships that could be construed as a potential conflict of interest.
